# Preschoolers' Precision of the Approximate Number System Predicts Later School Mathematics Performance

**DOI:** 10.1371/journal.pone.0023749

**Published:** 2011-09-14

**Authors:** Michèle M. M. Mazzocco, Lisa Feigenson, Justin Halberda

**Affiliations:** 1 Kennedy Krieger Institute, Baltimore, Maryland, United States of America; 2 Department of Psychiatry and Behavioral Sciences, Johns Hopkins University, Baltimore, Maryland, United States of America; 3 Department of Interdisciplinary Studies, Johns Hopkins University, Baltimore, Maryland, United States of America; 4 Department of Psychological and Brain Sciences, Johns Hopkins University, Baltimore, Maryland, United States of America; Yale University, United States of America

## Abstract

The Approximate Number System (ANS) is a primitive mental system of nonverbal representations that supports an intuitive sense of number in human adults, children, infants, and other animal species. The numerical approximations produced by the ANS are characteristically imprecise and, in humans, this precision gradually improves from infancy to adulthood. Throughout development, wide ranging individual differences in ANS precision are evident within age groups. These individual differences have been linked to formal mathematics outcomes, based on concurrent, retrospective, or short-term longitudinal correlations observed during the school age years. However, it remains unknown whether this approximate number sense actually serves as a foundation for these school mathematics abilities. Here we show that ANS precision measured at preschool, prior to formal instruction in mathematics, selectively predicts performance on school mathematics at 6 years of age. In contrast, ANS precision does not predict non-numerical cognitive abilities. To our knowledge, these results provide the first evidence for early ANS precision, measured before the onset of formal education, predicting later mathematical abilities.

## Introduction

The Approximate Number System (ANS) is a mental system of magnitude representations that produces an intuitive “number sense” across species [1] and throughout human development, starting from just after birth [2]. ANS representations are formed in response to visual, tactile, and auditory stimuli, and are activated whenever a person perceives or thinks about quantities [3], such as when gauging which of several containers has more berries, irrespective of the size of individual berries.

Unlike numerical skills targeted by formal schooling, magnitude representations of the ANS are independent of symbolic representations such as numerals (e.g., *4* or *7*) or number words (e.g., *four* or *seven*), as evidenced by their presence in non-verbal and pre-verbal populations [1], [3], [4]. Critically, unlike symbolic integer representations, ANS representations are inherently “noisy.” Whereas integer representations allow a thinker to distinguish small differences between quantities (e.g., to distinguish 104 berries from 105 berries via precise verbal counting), ANS representations are imprecise estimates that will often fail to support fine-grained numerical distinctions. For example, an adult using the ANS may correctly decide that a container with 105 berries has more than another container with 65 berries; but may fail to determine that a container with 105 berries has more than one with 95 berries – these quantities are too close to reliably distinguish without counting.

What is the relationship between the intuitive number sense that is supported by the ANS and more formal mathematical abilities? Recent investigations of individual differences in ANS representations suggest corresponding differences in mathematical ability. Individual differences in the ANS have been measured in terms of differences in the precision of people's approximate number representations. When a person views an array of items (e.g., 105 berries) too quickly to count, an ANS representation (e.g., “approximately one hundred”) is activated. The noise surrounding this estimate is large for numbers of this magnitude because the degree of error in the ANS representation increases linearly as the number being represented increases. This leads to ratio-dependent performance on numerical discrimination tasks (such as judging which of two briefly presented arrays is more numerous), in accord with Weber's Law [5]. The amount of noise in ANS representations varies, both across development and across individuals. Developmentally, gains in the precision of the ANS are reflected by changes in the numerosities that observers can reliably discriminate, such that, over time, children succeed when comparing arrays instantiating increasingly more difficult ratios (e.g., 1∶2 vs. 2∶3, then 3∶4, etc.). These gains have been observed in cross-sectional studies of infants [6], three- to six-year-old children, and adults [7], [8]. Furthermore, even within a single age group, large individual differences in numerical acuity have been observed [9].

Critically, individual differences in the ANS appear to be linked to mathematics ability. For example, we have shown that the precision of ninth graders' ANS representations retrospectively correlates with their standardized mathematics achievement scores obtained up to 8 years prior (i.e., at kindergarten) [9]. These associations persist when statistically controlling for task-specific cognitive skills and estimates of full-scale intelligence (FSIQ). More recently, Gilmore and colleagues [10] reported an association between kindergarteners' ability to perform approximate addition problems based on briefly shown arrays (e.g., mentally adding 9 dots and 6 dots, then judging whether the sum was more or less than 12 dots) and mathematics performance measured two months later, controlling for effects of verbal IQ.

What drives the reported relationship between ANS precision and math ability? Two competing hypotheses propose that the ANS either underlies, or is itself refined by, formal mathematical learning. If the ANS underlies formal mathematical ability in childhood, it may be a fruitful target for early instruction and intervention (as proposed by Wilson and colleagues [11]). Moreover, if ANS skills are predictive of a child's later school mathematics ability, they may be useful for screening children at risk for poor mathematics achievement or for identifying children prone to high achievement. In contrast, if refinement of the ANS is merely a reflection of the quality of instruction a child has received, then fine-tuning the ANS earlier in development may not be as useful.

These two hypotheses are not mutually exclusive. The ANS and formal mathematical ability might support and refine one another, in both directions, across development. One way of determining the initial state of their relationship is to ask whether ANS skills measured prior to schooling (i.e., prior to differences in the quality of children's formal mathematics instruction) predict achievement levels attained after the onset of formal math instruction. To date, the few studies of numerical ability predicting mathematics achievement have not relied on pure measures of the ANS as predictors, and have been carried out with children already enrolled in school. These studies show that symbolic number skills such as verbal magnitude comparison predict later math achievement. For instance, counting, reading or writing numerals, and symbolic magnitude comparisons measured at kindergarten predict math achievement at Grade 2 [12] or Grade 3 [13]; and symbolic number skills at Grade 1 predict math achievement at Grade 2 [14]. In a recent longitudinal study, children's magnitude comparisons of non-symbolic and symbolic quantities measured at kindergarten predicted their math ability at Grades 1 and 2 [15]. However, the non-symbolic comparison task used in that study did not involve fixed display times, and therefore may have measured verbal counting rather than ANS representations. Moreover, when the initial testing occurs during or after kindergarten, children are likely to have already received formal instruction in number symbols and operations, as reflected in published standards for school mathematics [16].

Here we assessed whether ANS precision measured prior to entering school predicts school mathematics during or after kindergarten. We first measured children's ANS precision at 3 to 4 years of age, using a nonverbal, non-symbolic comparison task; we then measured the same children's mathematics abilities two years later. We found that children's ANS precision measured at preschool, prior to formal instruction in mathematics, selectively predicted their school mathematics performance at age 6.

## Materials and Methods

### Ethics Statement

The research procedures described below were completed in accordance with approval from the Institutional Review Board at the Johns Hopkins University. Written consent was obtained from parents of all participants prior to testing.

### Participants

We tested 17 children (7 girls, 10 boys) who, at preschool, had participated in a cross-sectional study of ANS precision in 3 to 6 year olds [7]. Two years later these children were invited to return for a follow up assessment if they were enrolled in kindergarten (n = 5), first grade (n = 11) or second grade (n = 1) at the time of follow-up recruitment. The children's mean ages at both assessments and the interval between testing sessions appear in Table 1.

**Table 1:** Participant ages at, and time intervals between, preschool and follow up assessments in years and months (N = 17).

Time Point	Mean	Std. Deviation	Age Range
Age at Preschool	4; 2	0; 4.5	3; 5 to 4; 11
Age at Follow up	6; 8	0; 4.2	6; 2 to 7; 5
Interval between assessments	2; 6	0; 2.8	2; 0 to 2; 9

Recruitment was conducted via mail and telephone. Most of the participants were white (n = 14), and all 17 had parents of middle socioeconomic status who had completed at least some higher education.

### Measures and Procedures

At both preschool and follow up testing, children were seen individually for one session, in the research lab. The assessment administered when participants were in preschool was limited to one measure. At follow up testing, several measures were administered, in a fixed order.

**Figure 1:**
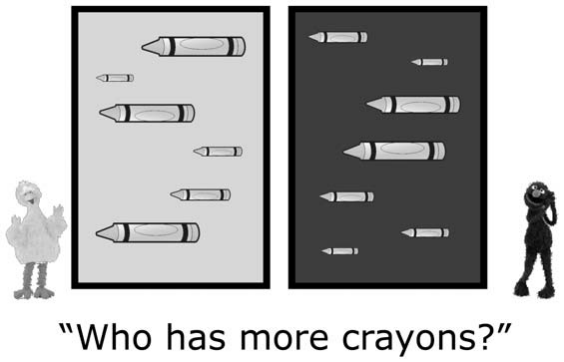
Sample trial used to estimate ANS precision as a function of the numerical ratio between arrays. We measured children's ANS precsion by having children judge whether Big Bird or Grover had more objects [e.g., crayons], with objects flashed too briefly to allow verbal counting.

We used performance on an ANS numerical discrimination task as our predictor measure of preschool ANS precision (described elsewhere in greater detail [7]). Briefly, children sat facing a large video screen on which two arrays appeared. Each array contained 1 to 14 images of identical familiar objects (e.g., blocks, crayons, wagons, etc.) of varying sizes that appeared within background frames demarcating “Big Bird's (objects)” and “Grover's (objects)” (see Figure 1). The examiner and parent or caregiver sat behind the child to avoid influencing performance. The testing began with a recorded female voice saying, “Let's play a game,” followed by four practice trials. On practice trials the computer first displayed Big Bird's objects accompanied by the phrase, “Here are Big Bird's [crayons].” Next, the computer displayed Grover's objects accompanied by the phrase, “Here are Grover's [crayons].” Finally, both arrays appeared simultaneously accompanied by the phrase, “Who has more [crayons]?” with label onset synchronized to the objects' visual onset. Children responded with a color-coded keyboard, pressing a yellow key to indicate that Big Bird had more objects and a blue key to indicate that Grover had more objects.

Several controls ensured that children remained focused on the number of objects throughout the task – as opposed to other dimensions, such as object size. Displays were controlled either for average object size (area correlated trials) or summed continuous extent (area anti-correlated trials). For each ratio presented, on half of the trials the larger numerosity had more total surface area (area correlated trials), and on the other half of trials the smaller numerosity had more total surface area (area anticorrelated trials). Area anticorrelated trials equated the total summed perimeter of Big Bird's and Grover's objects and anticorrelated their total surface area, two dimensions of continuous extent to which infants have shown sensitivity [17], [18]. Individual object sizes also varied within each display on each trial (Figure 1). In this way, only responses based on the number of objects would result in accurate performance throughout the task.

Sixty-six test trials followed. These were structured just like the simultaneous portion of the practice trials: Big Bird's and Grover's objects appeared simultaneously (synchronized to the phrase, “Who has more [crayons]?”) and remained visible for a fixed interval (either 1200 (n = 12) or 2500 ms (n = 5) depending on children's age at testing). After the objects disappeared, the images of Big Bird, Grover, and the empty background frames remained onscreen until children responded.

Each trial displayed numbers of objects drawn from a wide range of numerical ratios. Unequal numbers of trials of each ratio were presented, in order to focus on the more difficult ratios. Each child was tested with two trials per ratio bin for the ratios 1∶2, 2∶3, 3∶4, and 4∶5, with ten trials per ratio bin for the ratios 5∶6, 6∶7, and 7∶8, and with 14 trials per ratio bin for ratios 8∶9 and 9∶10, with the absolute number of objects in each array ranging from 1 to 14. (Trials of the same ratio could include different absolute numbers of objects (e.g., 5∶10 and 7∶14)). Across trials, the ratio, number of objects within each array, and object type varied randomly for each child, with the restriction that each child received the same number of trials from each ratio. A recorded voice provided positive or negative feedback after a child responded. The entire procedure lasted approximately 5 minutes.

Measures of ANS precision can be derived for groups or individual participants. By combining all subjects into a single group, accuracy on this ANS task as a function of ratio can be modeled psychophysically to determine the most difficult ratio that still results in accurate discrimination (i.e., the Weber fraction, *w*). The current sample of 17 children contributed to this type of group analysis and psychophysical modeling, as reported elsewhere [7]. In contrast, in the present study we focused on measuring individual differences in children's ANS precision. This required an alternative approach, because performance of the psychophysical model was quite volatile when fitting data from individual subjects (who were tested on only a few trials in which easier ratios were presented). Therefore, we focused on each child's percent of correct responses across the different ratios bins as our variable of interest. A Weber fraction (*w*) is a psychophysical description of how a child's percent correct should change with ratio. As such, when using percent correct as a less volatile proxy for *w*, it is critical that every child be tested on same ratios, because the total percent correct would be compromised if some children received a higher percentage of problems with harder ratios relative to other participants. Our method ensured that every child received the same ratios and the same numbers of trials within each ratio. For this reason, total percent correct in our task can be used as a less volatile proxy for *w*. In later work [19] we have determined the number of trials, ratios and display times that reduce the volatility of *w* for children of this age (see also, www.panamath.org).

When children returned at 6 years of age, we used standardized tests to assess their mathematical and general cognitive abilities. First, we administered the Test of Early Mathematics Ability – Third Edition (TEMA-3 [20]) as our primary outcome variable. We selected the TEMA-3 because in our earlier work we showed that TEMA-3 performance captures more variability in mathematics performance among preschoolers relative to other standardized measures of mathematics [21]. The TEMA-3 is normed for use with children ages 3 to 8 years. Typical TEMA-3 items administered to 6-year-olds involve counting, reading or writing two-digit numbers, adding or dividing quantities with manipulatives, determining the relative magnitude of symbolic numbers, symbolic arithmetic facts, evaluating addition number sentences, and mental addition with one-digit addends. Test-retest reliability for the TEMA-3 is .93 [20]. Our variable of interest was the age-referenced normative score, based on a mean of 100 (SD = 15).

We used the Wechsler Abbreviated Scale of Intelligence (WASI [22]) to measure global cognitive abilities. The WASI is normed for use with children and adults and includes verbal and nonverbal subtests. The Vocabulary subtest is a measure of expressive vocabulary and verbal knowledge; in general, vocabulary subtests are considered relatively good estimates of general intelligence [23]. The Block Design and Matrix Reasoning subtests are measures of perceptual organization and spatial reasoning abilities. Block Design, which also involves visual-motor coordination, requires reproducing two-dimensional geometric patterns under time constraints. Matrix Reasoning, an untimed measure of classification of visual patterns, requires nonverbal reasoning ability to identify the missing portion of a matrix grid from an array of choices. Each WASI subtest yields an age-referenced standard score that contributes to an overall FSIQ score. Short-term (2–12 weeks) reliability for WASI subtest scores are good, with reported stability coefficients in children ranging from .76 to .85 [22]. For each subtest, our outcome variable of interest was an age referenced T score (mean = 50, SD = 10).

Finally, we administered three subtests of the Rapid Automatized Naming (RAN) test, a timed measure of lexical retrieval [24], to distinguish processing of numerical and non-numerical stimuli. During the RAN, children name stimuli that appear in a linear 5×10 array, from left to right, as quickly as possible. The examiner measures children's speed to report fifty instances of single letters (a, d, o, p, s), colored squares (blue, green, yellow, red, black), or numbers (2, 4, 6, 7, 9) presented in varying order. For each subtest, our outcome variable of interest was response time (RT) in seconds, measured with a hand-held stopwatch.

We hypothesized that ANS precision would predict formal mathematics skills (TEMA-3), but not other aspects of cognitive performance. Hence, we predicted significant associations between ANS precision and the TEMA-3, but not the WASI subtests. Moreover, we hypothesized that ANS precision would be associated with only the Numbers subtest of the RAN.

## Results

### Preliminary Analyses

**Figure 2:**
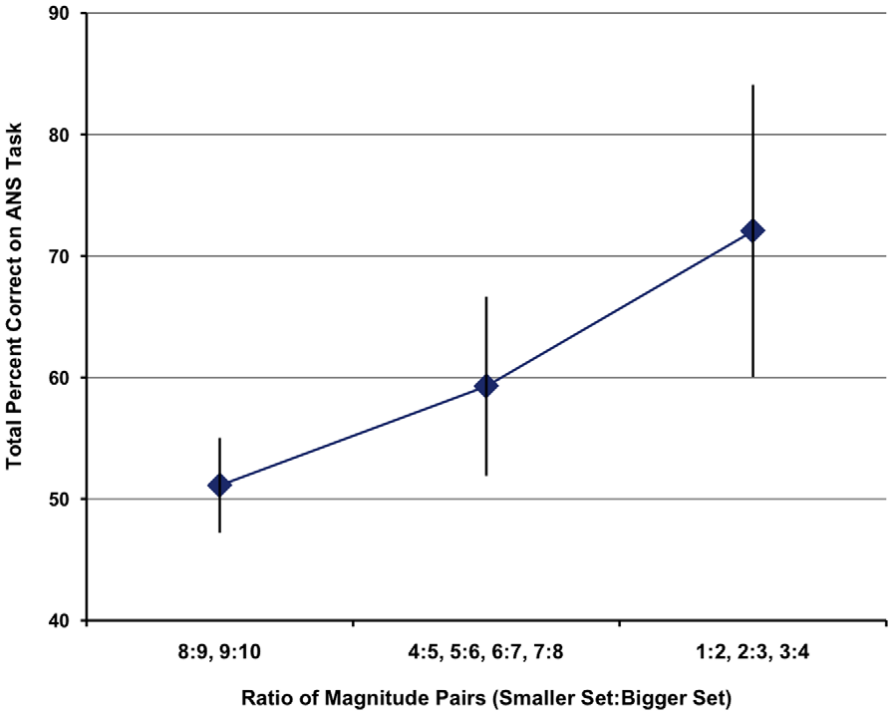
Group means for preschoolers' numerical discrimination performance as a function of the size of the numerical ratio between item arrays. Group mean values correspond to percent correct scores for three levels of ratio size: small, intermediate, and large. Error bars are 95% confidence intervals.

We first evaluated performance on the ANS numerical discrimination task at preschool, in order to verify that the task engaged children's ANS. Collapsing across all trials, percent correct scores ranged from 43% to 82% (Mean = 61.09%, SD = 11.14), and were normally distributed. As anticipated, when examined across three levels of ratio size – small (8∶9 and 9∶10), intermediate (4∶5, 5∶6, 6∶7, and 7∶8), and large ratios (3∶4, 2∶3, and 1∶2) – the mean Percent Correct score increased with ratio size, consistent with Weber's Law (Figure 2). Descriptive statistics for this predictor variable at preschool, and all outcome variables at follow up testing, appear in Table 2.

**Table 2:** Performance on predictor and outcome variables for the total study sample (N = 17).

Measure	Mean	Std. Deviation	Range
Preschool Numerical Discrimination (Percent Correct)	61.09	11.14	43.27–82.25
TEMA – 3 (Standard Score)	114.12	8.57	98–130
Vocabulary (T score)	59.88	5.86	50–69
Block Design (T score)	54.71	10.90	40–80
Matrix Reasoning (T score)	63.18	13.45	37–79
RAN Color (RT in seconds)	35.02	8.17	25.23–53.42
RAN Letter (RT in seconds)	27.24	8.46	16.41–45.40
RAN Number (RT in seconds)	24.06	6.30	15.32–37.52

Despite the overall improvement in performance accuracy as ratio size increased, four preschoolers performed quite poorly overall, scoring at or near chance on the ANS task (≈50%). Although it is possible that these children engaged a strategy independent of the ANS (e.g., they may have guessed), it is also possible that they were more numerically challenged than their peers, even when faced with arrays conforming to a 2∶1 ratio. As representatives of preschoolers with the least precise ANS skills, their inclusion in the subsequent analyses is important; however, if indeed these children were not engaging ANS supported skills, their exclusion is warranted. Since it is unclear which of these two explanations accounts for these children's poor performance, the subsequent sets of analyses were first conducted with the entire sample of 17 children, and then were repeated without those children performing at chance. Descriptive statistics for this latter subgroup are reported in Table 3.

**Table 3:** Age and scores on predictor and outcome variables among children performing above chance on the ANS task (N = 13).

Measure	Mean	Std. Deviation	Range
Age at Preschool, in Years, Months	4; 2	0; 5	3; 5–4, 11
Age at Follow up, in years, months	6; 8	0; 4	6; 2–7; 5
Preschool Numerical Discrimination (Percent Correct)	65.10	9.42	55.54–82.25
TEMA – 3 (Standard Score)	113.92	9.11	98–130
Vocabulary (T score)	59.23	5.69	50–67
Block Design (T score)	53.15	10.38	40–80
Matrix Reasoning (T score)	62.69	14.06	37–79
RAN Color (RT in seconds)	35.99	8.78	25.23–53.42
RAN Letter (RT in seconds)	27.06	8.86	16.41–45.40
RAN Number (RT in seconds)	22.50	5.93	15.32–37.52

### Primary Analyses

We used three sets of linear regression models to address our primary research questions. In each case, ANS precision at 3- to 4-years of age, indexed as the total Percent Correct on the numerical discrimination task, was entered as the predictor variable. Stimulus display times for this task varied across participants as a function of age, so in all analyses we calculated residual scores to adjust for age and display times at preschool testing.

**Figure 3:**
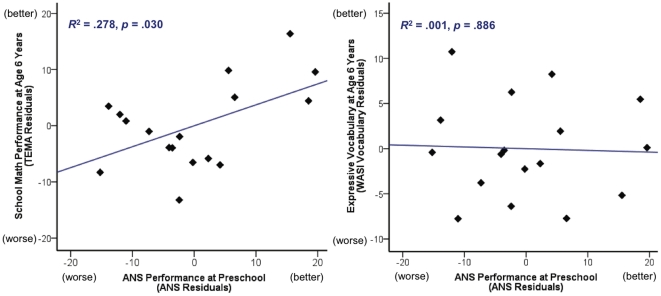
Associations between ANS accuracy (total percent correct scores adjusted for display time and age at testing) and either mathematics or language scores (each adjusted for age and grade at testing). For all three measures, higher scores indicate better performance.

Our first question was whether ANS precision at preschool predicts school mathematics performance. We conducted a linear regression analysis to evaluate the prediction of TEMA-3 scores (adjusted for age and grade at follow up testing) from the total Percent Correct score on the preschool ANS numerical discrimination task (adjusted for age and display time at initial testing). The model was significant, with ANS precision accounting for 28% of the variance in TEMA-3 score, *r^2^* = .278, *t* (16) = 2.405, *p* = .030 (Figure 3). This demonstrates an association between ANS precision prior to schooling and mathematics performance after the onset of formal instruction. As an indication of the strength of this association, even the concurrent measure of FSIQ was less predictive of TEMA-3 score than was preschool ANS; when evaluated alone, FSIQ at primary school accounted for approximately 7% of variation in concurrent TEMA-3 performance *r^2^* = .068, *p* = .312.

This pattern of findings held when the analyses were limited to the children whose ANS performance exceeded chance levels. For these 13 children, ANS precision at preschool (adjusted for age and display time at testing) accounted for 35% of the variance in TEMA-3 performance at age 6 years (adjusted for age and grade at testing), *r^2^* = .354, *t* (12) = 2.456, *p* = .032. FSIQ did not predict concurrent TEMA-3 score in this subgroup, *p* = .430.

As aforementioned, although the traditional index of ANS precision in our work and that of others has been the Weber fraction score (*w*), the volatile fits of our preschoolers' individual performance by the psychophysical model made *w* a less useful index of our subjects' performance [8]. However, despite this disadvantage, when we examined the relationship between *w* and later mathematics performance we found that individual *w* scores for the 14 participants whose data conformed to the psychophysical model, adjusted for age and display time at testing, accounted for 21% of the variance in TEMA-3 performance, *r* ^2^ = .208. Although this did not reach statistical significance, *p* = .10, this finding was in the predicted direction.

The predictive value of early ANS precision on later school mathematics may reflect a specific relationship between intuitive and formal numerical tasks, or it could just reflect an association between earlier and later cognitive skills. To test this we asked whether ANS precision at preschool predicted scores on any of the WASI subtests. For each subtest we conducted a linear regression analysis with WASI subtest score as the outcome variable (adjusted for age and grade at testing), as predicted by the preschool ANS task percent correct score (adjusted for age and display time). ANS precision was not a significant predictor of Vocabulary at 6 years, *r^2^* = .001, *p* = .886 (Figure 3); nor was it a significant predictor of Block Design or Matrix Reasoning performance, *p*s>.21. Similarly, for the subgroup of 13 participants who were above chance on the ANS task, ANS precision did not predict WASI Vocabulary, *p* = .445, nor the two remaining subtests, *p*s>.079.

**Figure 4:**
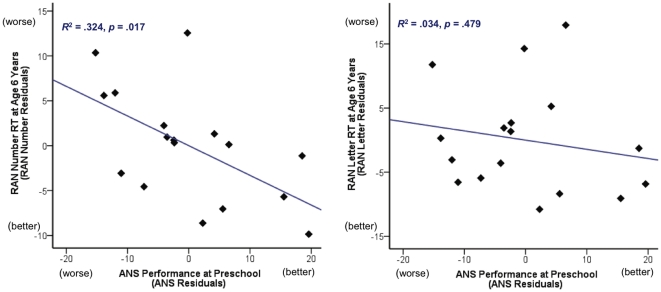
Associations between ANS accuracy (total percent correct scores adjusted for display time and age at testing) and RAN Number or Letter subtest reaction times (adjusted for age and grade at testing). For ANS performance, higher scores indicate better performance. For RAN response times (RT), higher scores indicate poorer performance.

As an additional test of specificity, we asked whether ANS precision at preschool (adjusted for age and display time) predicted response time on RAN Numbers, but not RAN Colors or RAN Letters, at primary school (controlling for age and grade at follow up testing). For each subtest we conducted a linear regression analysis with ANS precision at preschool as the predictor and RAN RT at primary school as the outcome. When RAN Colors or Letters subtest response times were included as the outcome variable, neither model was significant, *r^2^*<.04, *p*>.46. However, as predicted, ANS precision was a significant predictor of RAN Numbers response time, *r^2^* = .324, *t* (16) = −2.680, *p* = .017 (Figure 4), although this pattern did not hold when limited to the smaller subgroup of 13 participants who performed above chance on the numerical discrimination task, all *p*s>.25.

## Discussion

This is the first study to show that ANS precision measured years prior to formal schooling predicts mathematics ability in primary school. This association is not explained by possible confounds of general full-scale IQ. It appears specific to mathematics, since no such association emerged for ANS precision and measures of expressive vocabulary (i.e., WASI), perceptual organization (i.e., Block Design, Matrix Reasoning), or non-numerical lexical retrieval (i.e., RAN Colors and Letters). Finally, the strength of the relationship we observed in this sample (*r^2^* = .278 for TEMA-3 and .324 for RAN Numbers, among the total sample) is comparable to the strongest retrospective association we found previously between ninth graders' ANS precision and their earlier mathematics achievement scores (*r^2^* = .324 [9]).

It is noteworthy that our findings emerged despite the relatively restricted range of average to above average TEMA-3 scores (98 to 130) obtained by our small study sample, particularly given the wider range of TEMA-2 scores reported in our earlier longitudinal study of 64 ninth graders (60 to 133 at Kindergarten, as reported elsewhere [9]). This suggests that an even stronger correlation between ANS and math performance might have emerged from the present study had our sample of primary school students been more representative of the full range of mathematics achievement outcomes observed in general education classrooms. Moreover, a stronger correlation at primary (versus secondary) school is consistent with evidence that while ANS skills are related to (indeed, predictive of) future and current mathematics abilities, additional factors also contribute to mathematics achievement. Further longitudinal studies are needed to evaluate whether and how mediators of the relationship between ANS skills and symbolic mathematics vary as a function of other child characteristics, and how ANS skills might predict not only math performance at a given time but also trajectories of growth in formal mathematics skills over development.

Although here and elsewhere [9], [17] we found evidence of ANS representations correlating with formal math ability, some studies have failed to find evidence of this relationship [25], [26]. Variations in features of the non-symbolic tasks used to probe the ANS may account for some of these discrepancies. For example, the stimulus duration, numerical ratios, and number of trials presented appear to affect the degree of precision with which individual subjects' ANS representations can be measured. More recently [19] we have determined the values of these variables that allow an accurate fit for the *w* parameter for three and four year olds (see also, www.panamath.org). An additional possible source of the discrepancy between our findings and previous null results is the mathematics achievement outcome measure used, which has ranged across studies from brief, timed arithmetic trials (e.g., mathematics fluency) to untimed tests of paper and pencil calculations, and from test items that tap more intuitive numerical judgments to items that require knowledge of formal notation (e.g., fractions). Further work is needed to delineate the relationships between the ANS, symbolic number skills, and differing components of mathematics achievement.

Finally, influences on mathematical learning other than the ANS and other “number sense” skills range from motivational factors [27], teachers' content knowledge and knowledge of pedagogy [28], teacher's [29] and students' mathematics anxiety [30], student-teacher relationships [31], curriculum and instruction [31], and domain-general cognitive skills including but not limited to working memory and processing speed [32], [33], [34]. This study extends this body of research on predictors of mathematics outcomes by identifying a foundational numerical ability – ANS precision – which may be a principle component of the mechanisms underlying mathematical learning.

If ANS skills influence mathematical ability, they may be important targets for early intervention or instruction and may even guide efforts to vary some aspects of mathematics instruction on the basis of individual students' foundational skills. Whether this proves to be the case depends on the nature of this association. Effective applications will require greater specification regarding the ways in which the ANS drives mathematical learning, whether its role is direct or indirect, whether its primary role is to support early symbolic instruction [35] or to mediate the influences of other factors on mathematical learning such as working memory or cognitive control [36], and whether the nature of the relationship between ANS and mathematical outcomes varies with development or child characteristics. Our findings do not counter other well-known predictors of mathematics outcomes, such as the well documented effects of impoverished learning environment [37], [38]. However, our findings do add to the growing body of evidence that individual differences in cognitive skills make a powerful contribution to children's mathematical learning.
